# Metformin induces lipid changes on sphingolipid species and oxidized lipids in polycystic ovary syndrome women

**DOI:** 10.1038/s41598-019-52263-w

**Published:** 2019-11-05

**Authors:** Irene Pradas, Susana Rovira-Llopis, Alba Naudí, Celia Bañuls, Milagros Rocha, Antonio Hernandez-Mijares, Reinald Pamplona, Victor M. Victor, Mariona Jové

**Affiliations:** 1 Department of Experimental Medicine, Lleida University-Institute for Research in Biomedicine of Lleida (UdL-IRBLleida), 25198 Lleida, Spain; 2 Foundation for the Promotion of Healthcare and Biomedical Research in the Valencian Community (FISABIO), Service of Endocrinology, University Hospital Dr. Peset, 46017 Valencia, Spain; 3 Fundación Investigación Hospital Clínico Universitario/INCLIVA, Valencia University, 46010 Valencia, Spain; 4 Department of Medicine, Valencia University, 46010 Valencia, Spain; 5 Department of Physiology, Valencia University, 46010 Valencia, Spain

**Keywords:** Lipidomics, Endocrine reproductive disorders

## Abstract

Metformin is one of the treatments used for PCOS pathology decreasing body weight, plasma androgen, FSH and glucose levels. Unfortunately, there is little known about metformin’s effects on lipid metabolism, a crucial process in PCOS pathology. We have employed a lipidomic approach to explore alterations in the plasma lipid profile of patients with PCOS following metformin treatment. The aim is to offer new insights about the effect of metformin in PCOS patients. Plasma samples were obtained from 27 subjects prior to and following 12 weeks of metformin treatment. A detailed biochemical characterization and lipidomic profile was performed. Metformin reduces BMI, HOMA-IR, FSH and androstenedione and increases DHEA-S but no changes were found in glucose levels after treatment. Multivariate statistics revealed a specific lipidomic signature due to the effect of 12 weeks of metformin treatment in PCOS patients. This signature includes changes in sphingolipid metabolism suggesting a crosstalk between these lipid species and the androgenic metabolism and a decrease in oxidized lipids reinforcing that metformin treatment improves oxidative stress status. Our study confirms the specific effect of metformin in lipid metabolism on women with PCOS after 12 weeks of treatment.

## Introduction

Polycystic ovary syndrome (PCOS) is a multifactorial disorder that affects 7–9% of women of a reproductive age, and is characterized by clinical/biochemical hyperandrogenism, polycystic ovarian morphology (during ultrasound) and chronic oligo-anovulation. It is a multifaceted disease in which uncontrolled ovarian steroidogenesis, excessive oxidative stress, aberrant insulin signaling, and genetic/environmental factors play a role^1^. In addition, androgen levels are an intrinsic factor^2^.

PCOS is a pathology specifically associated with insulin resistance (IR) in which both the receptor and the mechanism of action of insulin are affected in different target tissues^3^. Although obesity seems to be heavily implicated in the pathogenesis of PCOS, affecting 40–70% of patients^4^, a considerable proportion of non-obese PCOS patients (30%) have IR^5^. Although the causal factor that leads to IR in PCOS has not been fully deciphered, a marked correlation between IR and an inappropriate accumulation of lipid species in insulin target tissues have been described. In line with this, there is evidence indicating that lipids act as signalling molecules and mediates IR^6,7^. IR is considered to underlie other aberrations that affect the health of PCOS patients^8^. For example, it influences lipid profile, with 70% of women with PCOS exhibiting at least one abnormal lipid constituent^9^. Furthermore, female PCOS patients with obesity often have dyslipidaemia, with higher triglyceride (TG) levels and lower high-density lipoprotein cholesterol (HDL-C)^10^ with respect to the healthy population. Studies have reported significantly lower levels of HDL-C in PCOS women versus weight-matched controls^11^. Approximately one-third of women with PCOS have been shown to also exhibit metabolic syndrome^12^. Indeed, PCOS is considered one of the ovarian manifestations of metabolic syndrome^13^. Alternatively, inflammation may also play a crucial role in the development of IR in women with PCOS^14,15^ and has previously been associated with other PCOS associated pathologies, including IR^16^. In relation to this, previous studies with PCOS patients have shown that acute hyperglycemia and IR enhance levels of proinflammatory transcription factor nuclear kappa B (NF-kB)^17^ and those of proinflammatory cytokines^17–19^.

In addition to hormonal derangements and altered glucose metabolism, PCOS has traditionally been linked with atherogenic dyslipidemia, a major determinant of cardiovascular diseases^20^. However, a recent study performed with PCOS patients without associated pathologies and presenting a non-pathogenic lipid profile (Total cholesterol, LDL-C, HDL-C and TG) describes the presence of a PCOS lipidomic fingerprint, in which glycerolipid, glycerophospholipid and sphingolipid metabolism is affected^21^, suggesting that these molecules are related to the physiopathology of PCOS and opening up new scenarios in the search for new drugs. Therefore, the lipid profile characterized by high triglycerides^22^, low HDL cholesterol and increased LDL cholesterol, which has been related to androgen excess^22,23^, is a classic profile linked to IR rather than PCOS. Further lipidomic studies that analyse 325 lipid species in women with or without PCOS show an association of BMI with 12 classes of lipid species including phospholipids, ceramides, gangliosides and acylglycerols, and a free androgen index with 8 classes of lipids, namely ceramides, phospholipids and acylglycerols, thus supporting prior findings that adiposity is a key driver of dyslipidaemia in PCOS^24^.

The underlying pathophysiological mechanisms of PCOS are not fully elucidated; consequently current therapeutic options for PCOS women mostly treat symptoms. Metformin is a biguanide used as the first choice oral anti-hyperglycemic drug to treat type 2 diabetes. Its mechanism of action is twofold: it promotes a reduction in glucose production and it improves insulin sensitivity, the latter being a consequence of changes in lipid metabolism^25^. The changes arise through the mitochondrial electron transport chain becoming inhibited by metformin and the consequent activation of 5′-AMP-activated protein kinase (AMPK), which inhibits fatty acid (FA) synthesis. In this way, metformin prevents lipid storage and the impairment of FA oxidation in insulin-sensitive tissues, both of which processes are described in IR^26^. Recently, metformin has also been used to management of hirsutism, acne and IR during PCOS^27,28^. Concerning IR, metformin exerts multiple actions on different insulin-sensitive tissues, including skeletal muscle, adipose tissue, liver, the endothelium and the ovaries^29^, and seems to improve the long-term health of PCOS women by prevent endometrial cancer, diabetes and cardiovascular disease^30^.

The expanding development of omics approaches has allowed a wider view of the molecular mechanisms underlying human pathology. Indeed, we have recently confirmed a specific plasma lipidomic signature in women with PCOS with respect to healthy individuals^21^. This signature implies changes in the metabolism of glycerolipids, glycerophospholipids and sphingolipids, pointing to changes in the glycerophospholipid biosynthetic pathway and cell signalling. Besides the documented role of metformin in lipid metabolism, the potential lipidomic changes in response to metformin treatment in PCOS patients has never been addressed and it would be interesting to determine if metformin can restore the previously reported lipidomic profile that is altered during PCOS^21^.

The present study aims to clarify whether metformin treatment in PCOS patients induces a specific lipidome profile in plasma, thus providing new insight into the management and treatment of this metabolic and endocrine disease.

## Results

### Anthropometric and metabolic parameters

Table 1 shows the anthropometric and metabolic parameters of our PCOS patients before treatment (pre-treatment, PRE) and after 12 weeks of metformin (post-treatment, POST). No statistical significance was found when comparing anthropometric parameters between groups for all the parameters except weight and BMI, significantly lower after 12-weeks of metformin treatment. Among subjects, 11 presented IR (HOMA ≥ 2.6, 2 overweight (BMI ≥ 25) and 12 obesity (BMI ≥ 30) before treatment. Parameters related to lipid metabolism (total cholesterol, LDL-c, HDL-c and triglycerides) were also similar in PCOS patients after metformin treatment. Glucose and insulin levels showed a trend to decrease after 12-weeks of metformin treatment. Although differences in glucose and insulin are not statistically significant, the HOMA-IR value is significantly lower after treatment with a reduction of 0.5 points (p < 0.05). We observed that the endocrine profile of PCOS subjects changed after 12 weeks of metformin treatment, as represented by decreased FSH and androstenedione levels and increased DHEA-S levels (p < 0.05 in all cases).

**Table 1:** Anthropometric, biochemical and endocrinological data of PCOS patients before treatment with metformin (pre-treatment, PRE) and at the end of 12-weeks follow-up (post-treatment, POST).

	PRE	POST	p-value B.H.
n	27	27	
Age (years)	24.7 ± 6.4	—	
Weight (kg)	78.9 ± 4.7	76.3 ± 4.4	**0.011**
BMI (kg/m^2^)	29.2 ± 1.8	28.2 ± 1.6	**0.010**
Waist circumference (cm)	94.9 ± 3.8	94.7 ± 3.4	0.882
SBP (mmHg)	119.5 ± 3.3	114.0 ± 3.2	0.100
DBP (mmHg)	73.4 ± 2.8	70.7 ± 2.1	0.220
Total cholesterol (mg/dl)	176.0 ± 5.8	172.1 ± 6.0	0.308
LDL-c (mg/dl)	112.5 ± 4.9	107.6 ± 5.3	0.106
HDL-c (mg/dl)	44.6 ± 2.1	44.7 ± 1.7	0.931
Triglycerides (mg/dl)	94.6 ± 11.6	101.1 ± 12.0	0.242
Glucose (mg/dl)	83.4 ± 1.9	79.5 ± 1.7	0.078
Insulin (μUI/ml)	14.05 ± 1.8	12.42 ± 1.4	0.071
HOMA-IR	2.97 ± 0.4	2.47 ± 0.3	**0.034**
FSH (mIU/ml)	4.89 ± 0.3	4.06 ± 0.3	**0.014**
LH (mIU/ml)	7.95 ± 2.3	5.63 ± 0.8	0.302
Free Androgen Index	0.79 ± 0.1	0.72 ± 0.1	0.814
Testosterone (ng/ml)	0.80 ± 0.1	0.73 ± 0.1	0.228
Androstenedione (ng/ml)	4.52 ± 0.4	3.70 ± 0.4	**0.013**
SHBG (nmol/l)	57.20 ± 8.5	45.25 ± 4.6	0.120
DHEA-S (µg/dl)	293.8 ± 49.8	335.69 ± 57.4	**0.027**

Data are expressed as mean ± SEM. Statistical significance (p < 0.05) was considered when compared by a paired t-test after Benjamini-Hochberg correction.

### Plasma lipidome of PCOS patients under metformin treatment

In order to assess how 12 weeks of metformin treatment effected the global plasma lipidomic profile of PCOS patients, we carried out a non-targeted lipidomic approach, focusing on low molecular-weight ionizable lipid molecules (m/z of 300–3000). In the case of both polarities (negative and positive), 1950 features were aligned after applying the MFE algorithm^31^. Then, we selected only those features present in at least 50% of the samples of the same group (312 lipid species ionized with positive polarity and 64 lipid species in negative polarity).

**Figure 1:**
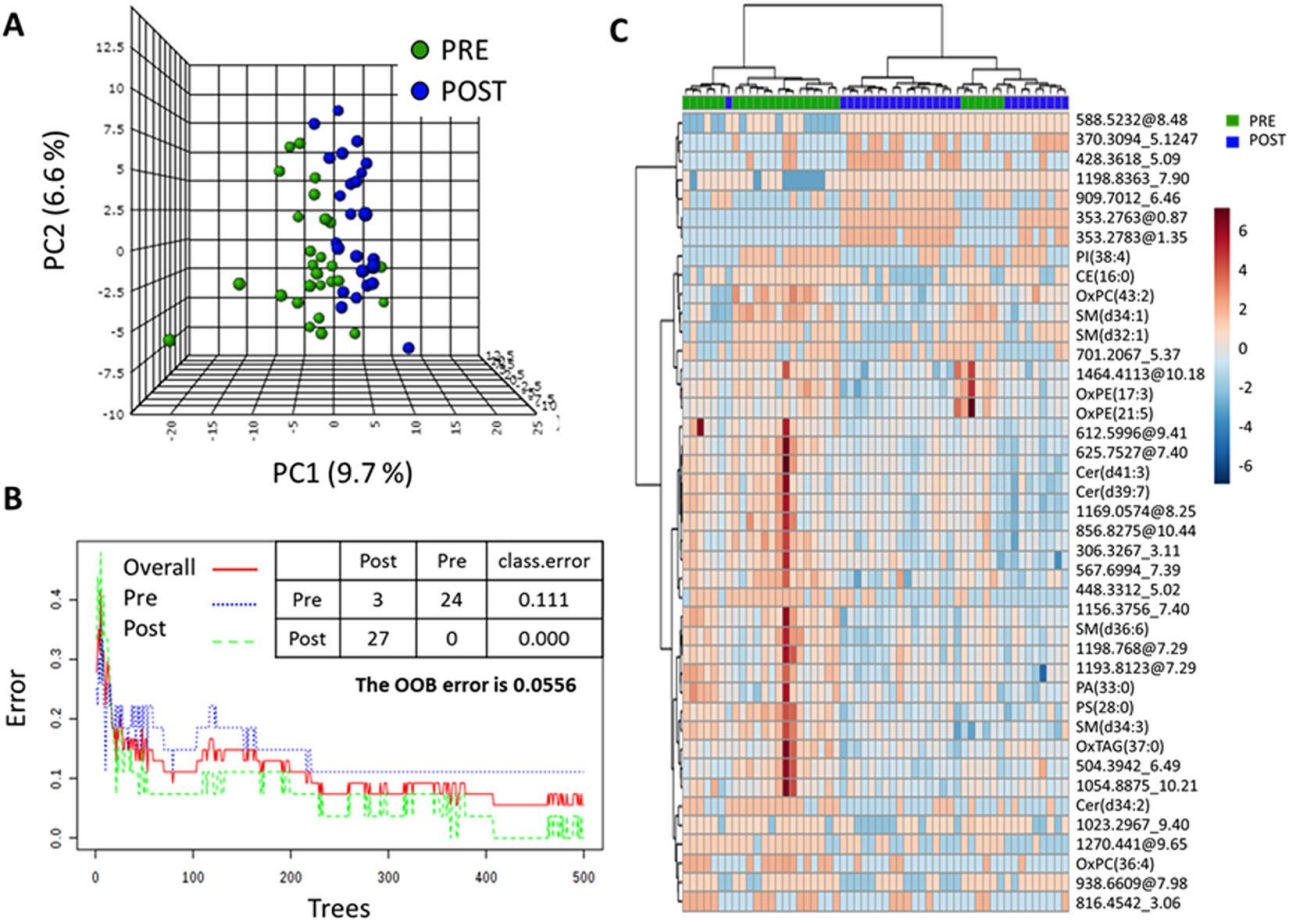
Multivariate statistics and machine learning show a specific lipidomic signature after 12-weeks metformin treatment in PCOS patients. **(A)** Unsupervised Principal Component Analyses (PCA) and (**B**) Random Forest (RF) classification indicate that it is possible to discriminate PCOS patients before (PRE) and after (POST) 12-weeks of metformin basing on their plasma lipidome. (**C**) Heat map representations of hierarchical clustering analyses using 42 most statistical significant lipids species (T-test Paired with Benjamini Hochberg Correction) between PCOS patients before and after 12-weeks metformin treatment. Unknown identities are represented as exact mass_ retention time. Metabolite identification is based on exact mass, retention time, isotopic distribution and/or MSMS spectrum. Each line of this graphic represents an accurate mass ordered by retention time, coloured by its abundance intensity normalized to internal standard and baselining to median/mean across the samples. The scale from −6 (blue) to 6 (red) represents this normalized abundance in arbitrary units. All the analyses have been performed with the 312 features detected and filtered in ESI positive polarity.

To determine whether the detected lipidome was altered in the PCOS patients after the 12-week of treatment multivariate statistics were applied (Fig. 1 and Fig. S1). All the analyses reflected in Fig. 1 were carried out with lipid species ionized with a positive ESI polarity, while the results of the multivariate analyses of lipidome with negative ESI are represented in Fig. S1. Non-supervised PCA (Fig. 1A) revealed clustering of both groups, thus suggesting that the effect of metformin in PCOS patients has a specific plasma lipidomic signature. In line with this, the machine learning algorithm Random Forest correctly classified all of the patients after the treatment (classification error PRE: 0.111, classification error POST: 0.000, out of bag error: 0.0556) based on the lipidome detected using the positive polarity mode (Fig. 1B). All of the multivariate analyses performed with the negatively charged lipid species showed no differences attributable to the metformin treatment in PCOS patients (Fig. S1).

To better characterize the effect of metformin on the plasma lipidome we performed a parametric paired t-test for equal variances. Of the 376 lipid species detected, 45 were found to differ in a statistically significant manner between groups. The hierarchical analysis of these 45 lipid species clearly showed a differential regulation after and before metformin treatment (Fig. 1C). Interestingly, all of the 18 identified lipid species (based on exact mass, retention time, isotopic distribution and/or MS/MS spectrum) were decreased after metformin treatment (Table 2), while the remaining 27 were unidentified (Table 3).

**Table 2:** Identified compounds statistically different between groups (paired t-test with Benjamini Hochberg correction).

Lipid Category	Compound	FC	p (BH Corr)	Change post treatment	AUC ROC	Specificity	Sensitivity	m/z	RT	Adduct
GL	OxTAG(37:0)b	−1.06	3.05E-02	down	0.68724	0.7	0.7	700.6212	9.89	M + NH4+
	OxPE(17:3)b	−1.40	5.85E-05	down	0.90123	0.9	0.9	536.1593	7.44	M + H+
	PA(33:0)a	−1.08	1.70E-03	down	0.79012	0.9	0.6	663.4474	7.97	M + H+
	PS(28:0)a	−1.04	4.74E-02	down	0.72702	0.7	0.7	680.4734	7.96	M + H+
GP	OxPC(43:2)b	−1.19	2.01E-03	down	0.71605	0.9	0.6	758.5637	7.63	M + H+
	OxPC(36:4)b	−41.39	1.99E-02	down	0.71811	0.9	0.5	812.5046	3.06	M + H+
	PI(38:4)c	−43.45	3.09E-02	down	0.71331	0.8	0.6	887.5621	6.35	M + H+
	PE(P-38:6)c	−54.66	4.47E-02	down	0.70096	0.9	0.6	746.5292	8.23	M + H+
	OxPE(21:5)b	−1.17	4.70E-02	down	0.83676	0.7	0.9	610.1774	7.93	M + Na+
	Cer(d34:2)a	−99.43	4.48E-03	down	0.74623	0.8	0.7	536.4794	6.05	M + H+
	Cer(d39:7)a	−1.12	3.48E-02	down	0.72291	0.9	0.6	596.5207	8.28	M + H+
	Cer(d41:3)a	−1.19	1.23E-02	down	0.8107	0.7	0.8	632.591	8.27	M + H+
SP	SM(d32:1)c	−65.34	2.32E-02	down	0.76818	0.7	0.7	675.5355	6.41	M + H+
	SM(d34:3)a	−1.12	1.65E-04	down	0.84088	0.9	0.7	699.5899	9.16	M + H+
	SM(d34:1)a	−1.54	8.25E-04	down	0.83813	0.9	0.7	703.5677	7.24	M + H+
	SM(d36:6)a	−1.08	8.69E-05	down	0.90672	0.9	0.8	721.5014	7.97	M + H+
	SM(d38:2)c	−1.06	1.16E-02	down	0.77778	0.9	0.7	757.6426	9.17	M + H+
ST	CE(16:0)a	−35.61	4.98E-02	down	0.738	0.8	0.7	642.6079	10.77	M + NH4+

^a^Lipid species identified by exact mass and retention time and MS/MS spectrum.

^b^Lipid species identified by exact mass and retention time and confirmed with LipidMatch libraries.

^c^Lipid species with a possible identity based on exact mass and retention time.

**Table 3:** Unidentified compounds statistically different between groups (Paired t-test with Benjamini Hochberg correction).

Compound	FC	p (BH Corr)	Change post treatment	mass	RT	ESI
307.3267_3.11	−1.22	1.70E-03	down	307.3267	3.11	POS
329.1986_2.74	−83.21	4.47E-02	down	329.1986	2.74	NEG
353.2763_0.87	33,497.97	2.03E-11	up	353.2763	0.87	POS
353.2783_1.35	4,901.52	6.69E-07	up	353.2783	1.35	POS
371.3094_5.12	201.58	9.70E-03	up	371.3094	5.12	POS
428.3618_5.09	258.04	2.93E-02	up	428.3618	5.09	POS
448.3312_5.02	−1.27	3.69E-04	down	448.3312	5.02	POS
504.3942_6.49	−1.09	9.70E-03	down	504.3942	6.49	POS
555.353_2.75	−210.03	1.01E-02	down	555.353	2.75	NEG
567.6994_7.39	−1.18	1.51E-02	down	567.6994	7.39	POS
588.5232_8.48	506.49	2.13E-03	up	588.5232	8.48	POS
612.5996_9.41	−1.15	9.70E-03	down	612.5996	9.41	POS
625.7527_7.39	−1.24	2.41E-03	down	625.7527	7.4	POS
701.2067_5.37	−32.21	4.45E-02	down	701.2067	5.37	POS
816.4542_3.06	−27.2	3.32E-02	down	816.4542	3.06	POS
856.8275_10.44	−1.16	8.16E-03	down	856.8275	10.44	POS
909.7012_6.46	62.93	1.96E-02	up	909.7012	6.46	POS
938.6609_7.98	−48.1	1.23E-02	down	938.6609	7.98	POS
1023.2967_9.4	−36.31	3.89E-02	down	1023.2967	9.4	POS
1054.8875_10.21	−1.09	3.32E-02	down	1054.8875	10.21	POS
1156.3756_7.4	−98.37	2.04E-02	down	1156.3756	7.4	POS
1169.0574_8.25	−1.31	2.13E-03	down	1169.0574	8.25	POS
1193.8123_7.29	−1.14	1.44E-02	down	1193.8123	7.29	POS
1198.768_7.29	−1.16	3.16E-03	down	1198.768	7.29	POS
1198.8363_7.9	32.52	1.16E-02	up	1198.8363	7.9	POS
1270.441_9.65	−234.77	3.97E-03	down	1270.441	9.65	POS
1464.4113_10.18	−1.41	5.74E-03	down	1464.4113	10.18	POS

NEG: negative ESI mode; POS: positive ESI mode.

Compounds are represented as exactmass_retentiontime.

Among the lipid species that could be identified, 8 belonged to the glycerophospholipid family, four of which (ethanolamine and choline GP-based on) were oxidized and one was a plasmalogen polyunsaturated species. We also identified 8 sphingolipids with a differential regulation due to the metformin treatment. Specifically, three polyunsaturated ceramides and five sphingomyelins, two of which were monounsaturated and three polyunsaturated. Finally, levels of one oxidized triacylglycerol and one cholesterol ester were also found to be lower after the treatment.

In order to further determine to what extent these metabolites can predict the effect of treatment of PCOS with metformin, ROC analyses were performed using MS peak areas. The details of the area under the curve, as well as the specificity and sensitivity of each lipid species, are presented in Table 2. The lipid species with the highest AUC were oxPE(17:3), with a value of 0.90123 and 90% specificity and sensitivity, and SM(d36:6), with 0.90672 AUC, 90% specificity and 80% sensitivity.

When plasma fatty acid composition was analysed, differences were found due to the effect of the 12-week metformin treatment (Table 4). There was a significant increase in the content of 20:2n-6, whereas 20:3n-6, 20:4n-6, 22:1n-9 and 22:5n-3 were found to have decreased. This led to a slight but significant alteration of the average chain length (ACL). When we evaluated other parameters based on fatty acid composition, such as SFA, UFA, MUFA, PUFA, PUFAn-3, PUFAn-6, DBI, PI, and AI, no differences were found in PCOS patients after the metformin treatment.

**Table 4:** Total fatty acid composition of plasma from subjects at the baseline and at the end of 12-weeks follow-up.

	PRE-TREATMENT	POST-TREATMENT	p-value
C14:0	0.668 ± 0.007	0.725 ± 0.009	0.23
C16:0	22.475 ± 0.06	22.614 ± 0.054	0.701
C16:1(n-7)	1.505 ± 0.019	1.456 ± 0.017	0.533
C18:0	8.103 ± 0.032	8.119 ± 0.044	0.927
C18:1(n-9)	20.948 ± 0.125	21.203 ± 0.137	0.641
C18:1(n-7)	1.647 ± 0.007	1.597 ± 0.006	0.257
C18:2(n-6)	31.644 ± 0.148	32.242 ± 0.177	0.497
C18:3(n-3)	0.285 ± 0.003	0.324 ± 0.005	0.169
C18:4(n-3)	0.144 ± 0.001	0.152 ± 0.001	0.11
C20:0	0.149 ± 0.001	0.182 ± 0.004	0.127
C20:1(n-9)	0.222 ± 0.002	0.237 ± 0.004	0.56
C20:2(n-6)	0.034 ± 0	0.054 ± 0.002	**0.042**
C20:3(n-6)	1.615 ± 0.015	1.472 ± 0.013	**0.015**
C20:4(n-6)	5.976 ± 0.046	5.597 ± 0.044	**0.006**
C20:5(n-3)	0.04 ± 0.001	0.048 ± 0.001	0.067
C22:0	0.253 ± 0.002	0.26 ± 0.002	0.505
C22:1(n-9)	2.074 ± 0.035	1.605 ± 0.019	**0.003**
C22:4(n-6)	0.177 ± 0.001	0.171 ± 0.001	0.077
C22:5(n-6)	0.143 ± 0.003	0.116 ± 0.001	0.114
C22:5(n-3)	0.251 ± 0.002	0.212 ± 0.002	**0.002**
C24:0	0.111 ± 0.002	0.099 ± 0.001	0.384
C22:6(n-3)	1.226 ± 0.016	1.176 ± 0.013	0.427
C24:1(n-9)	0.268 ± 0.002	0.262 ± 0.002	0.514
C26:0	0.039 ± 0.001	0.076 ± 0.006	0.286
ACL	17.845 ± 0.004	17.811 ± 0.003	**0.012**
SFA	31.798 ± 0.067	32.075 ± 0.071	0.527
UFA	68.202 ± 0.067	67.925 ± 0.071	0.527
MUFA	26.665 ± 0.133	26.36 ± 0.141	0.639
PUFA	41.537 ± 0.149	41.565 ± 0.176	0.974
PUFA(n-3)	1.947 ± 0.015	1.912 ± 0.015	0.586
PUFA(n-6)	39.59 ± 0.141	39.653 ± 0.175	0.941
DBI	130.443 ± 0.263	128.959 ± 0.278	0.282
PI	73.754 ± 0.31	71.893 ± 0.287	0.138
AI	48.762 ± 0.304	49.009 ± 0.31	0.862
Ratio MUFA/SFA	0.841 ± 0.005	0.822 ± 0.004	0.394

Abbreviations: ACL, average chain length; SFA, saturated fatty acids; UFA, unsaturated fatty acids; MUFA, monounsaturated fatty acids; PUFA, polyunsaturated fatty acids; PUFAn-3, polyunsaturated fatty acids series n-3; PUFAn-6, polyunsaturated fatty acids series n-6; DBI, double bond index; PI, peroxidizability index; AI, anti-inflammatory index.

## Discussion

PCOS is a heterogenic and multigenic metabolic disorder which affects women of a reproductive age and is characterized by hyperandrogenism and is commonly associated with other pathologies such as IR and obesity^32^. Currently, there is no universal treatment for PCOS patients, and most available drugs are directed at treating symptoms such as androgen excess, IR or oligo-ovulation^32,33^. Among available insulin-sensitizer drugs, metformin is one of the most used, having similar effects as lifestyle changes in terms of decreasing body weight/BMI and more marked effects in terms of decreasing androgen concentrations^34^. Furthermore, previous studies have demonstrated that metformin reduces plasma glucose levels and FSH in PCOS subjects, and also improves the oxidative stress status of patients, among other benefits^18^. By decreasing gluconeogenesis and lipogenesis and enhancing glucose uptake in the liver, skeletal muscle, adipose tissue and ovaries metformin increases insulin sensitivity. One potential mechanism proposed for the glucose-lowering effect of metformin is the inhibition of mitochondrial complex I and the consequent stimulation of AMP kinase^18,25,34^. On the other hand, the intracellular lipid-lowering effects of metformin have been associated with an increase in the mitochondrial channelling of fatty acids. Treatment with metformin enhances the mitochondrial β-oxidation process, directing excess intracellular FA towards β-oxidation, which reduces the supply of substrates for the synthesis of bioactive lipids, which in turn affects the insulin signalling pathway^35^. Furthermore, metformin blocks increments in the fatty acid transport protein CD36 and in aberrant ceramide and diacylglycerol content in the skeletal muscle of diabetic rats^36^. However, the molecular mechanisms through which metformin induces these changes are not fully understood. As far as we know, the effect of metformin on plasma lipid profile in PCOS has been basically focused on the study of lipoprotein profile. Therefore, the objective of this study was to characterize how a 12-week metformin treatment affects the plasma lipidomic profile of women with PCOS.

Anthropometric and metabolic parameters in PCOS patients prior to and following 12 weeks of metformin treatment showed a decrease in BMI values, whereas no changes in total triglycerides, total cholesterol, LDLc and HDLc levels were detected. This reduction in body weight could be partially responsible for the lipidomic changes in plasma described after metformin treatment. In terms of insulin resistance parameters, we found a slight decrease in plasma glucose and insulin levels after treatment, though these changes were not statistically significant. However, the HOMA-IR index decreased after the 12-week treatment, thus indicating an improvement in insulin resistance. These results are in line with most previously published studies on the subject and confirm the beneficial effect of metformin on body weight and insulin sensitivity^25^.

Endocrine measurements revealed several rearrangements after metformin treatment; namely, decreased levels of FSH and androstenedione and increased levels of DHEA-S. The FSH levels found in plasma patients after treatment were very similar to those detected previously in non-PCOS women, suggesting a reversion of PCOS pathology after metformin treatment^21^. With respect to androstenedione levels, after treatment we found very similar levels to those detected in “pure” PCOS women, suggesting that this parameter is affected by PCOS-associated pathologies^21^. Total testosterone concentrations are very much influenced by SHBG concentration, as 65% of plasma testosterone is bound to SHBG^37^. Previous studies in PCOS patients without associated pathologies showed no changes in SHBG and testosterone levels compared with control subjects, thus reinforcing the idea that PCOS-associated pathologies could be partially responsible for the changes observed in androgenic metabolism^21^. In the present study, we did not observe differences in testosterone and SHBG levels after metformin treatment.

Once we characterized the classical parameters of PCOS patients, we analyzed the whole plasma lipidomic profile using an LC-MS-based technique. Furthermore, fatty acid composition was also measured in order to better characterize the composition of the plasma lipidome. The results revealed the existence of a plasma lipidomic profile specifically associated with a 12-week metformin treatment in PCOS patients, which is the first time this has been reported. This signature is defined mostly by glycerophospholipids and sphingolipids; we detected 45 lipid molecular species whose concentration was altered by the effect of metformin treatment (7 lipid species were up-regulated, while 38 were down-regulated).

The interaction between sphingolipids and steroid hormones has previously been described, and this interaction modulates the steroidogenic signaling pathway. Specifically, these lipids can modulate steroidogenesis, acting at different levels as a second messengers, paracrine/autocrine regulators and/or ligands for nuclear receptors^38^. For example, ceramides have been shown to regulate progesterone and testosterone production, although the precise molecular mechanisms underlying this process are unclear and require further study^39^.

In the present work, we described lower levels of 3 ceramides and 4 sphingomyelins after metformin treatment, suggesting that the effect of metformin on androgenic metabolism is partially mediated by sphingolipid metabolism regulation. Moreover, the role of sphingolipids, and specially ceramides, in the mediation of insulin resistance^40–42^ has been reported previously, so the effect of metformin on parameters of insulin resistance in women with PCOS could also be a result of the interaction of these species with the insulin signaling pathway.

Concerning glycerophospholipids, we identified 8 species that were down-regulated after metformin treatment, 4 of which were oxidized. Surprisingly, we also identified an oxidized TAG. The effect of metformin on cell redox status has been described previously^18^. The present results reaffirm the improvement of oxidative stress in PCOS patients after metformin treatment and reveal that an improvement also takes place in the lipid metabolism, thus decreasing lipoxidative damage.

Globally, fatty acid analyses indicated minor changes in fatty acid composition after metformin treatment. Although we observed changes in C20-based polyunsaturated fatty acids (C20:2 (n-6), C20:2 (n-6), C20:6 (n-6)), C22:1 (n-9) and C22:5(n-3), the variations only represented about 0.2% of ACL reduction, while no changes were detected in the other parameters calculated. The present results may indicate a modulation of elongase and desaturase activity, especially in the elongases ELOVL3, 5 and 2 and desaturases Δ8 and 5^43^. Fatty acid elongation and desaturation are crucial processes in the biosynthesis of saturated, monounsaturated and polyunsaturated fatty acids. Furthermore, the deregulation of these enzymes’ activity has been previously related to IR and diabetes^44,45^. Specifically, previous studies in mice have reported a relationship between low ELOVL5 activity in the liver and glucose intolerance and insulin resistance^46,47^. In line with this, our results suggest that the minor alterations found in some PUFA could have been a response to slight changes in insulin levels and insulin sensitivity induced by metformin.

The principle limitation of the present study is the number of patients (n = 27) and the fact that they came from a reduced geographical area. Furthermore, we have used the Rotterdam criteria for this study, and not the last international guideline, due to the fact that our patients were part of a previously recruited cohort. Moreover, as we have stated before, the PCOS condition is usually associated with other pathologies, such as IR or obesity. In the present study we included 11 IR patients and 6 non-IR patients, and so we cannot be sure that IR was not a cofounding factor. Further studies with a larger cohort would guarantee a “purer” PCOS population. In addition to these population and clinical factors, our study has not enabled us to unravel the biological significance of this compositional complexity. Moreover, although sampling was scheduled to minimize the potential influence of diet on the lipidome, we cannot rule out the possibility that some compounds appearing as a result of the metabolism of nutrients affected the plasma lipidome in general, although the fact that our cohort was from a small geographic area would suggest that they were relatively homogeneous in terms of lifestyle and dietary habits.

All in all, we can conclude that metformin treatment induces a specific plasma lipidomic profile in PCOS women that is characterized mainly by a decrease in sphingolipids and glycerophospholipids and a reduction of lipoxidative species. We believe that this work could aid future research in the exploration of the molecular mechanisms involved in sphingolipid – steroid interaction. Our study confirms that lipid profile highlights a specific effect of metformin on PCOS women after 12 weeks of treatment.

## Methods

### Subjects

This study was carried out in the Service of Endocrinology at the University Hospital Dr. Peset (Valencia, Spain). Plasma samples of twenty-seven women with PCOS before and after treatment for 12 weeks with metformin were analysed. Following the criterion of our Hospital and according to a previous study published^18^ treatment with metformin was initiated at 500 mg per day (during the first 2 weeks). After 2 weeks it was increased to 1000 mg/day (weeks 3 and 4) and then to 1500 mg/d during weeks 5–12. Patients did not take any other medication.

Diagnosis of PCOS was confirmed using the Rotterdam criteria^48^. In brief, presence of oligoovulation (cycles longer than 35 days or less than 26 days)^49^; free testosterone levels higher than 0.5 ng/dl (this cut-off level was estimated as the mean ± 2 SD according to the levels in healthy women); hirsutism (Ferriman-Gallwey score > 7) and polycystic ovaries (presence of at least 12 small −2 to 9 mm- follicles in each ovary), assessed by trans-vaginal ultrasonography. Ultrasound scans were performed and scored independently by one of two experienced and blinded reviewers.

None of the subjects had any condition affecting her reproductive physiology or any systemic or endocrine disease or galactorrhea. Exclusion criteria were malignant neoplasia, active infectious diseases, anemia, diabetes mellitus, history of ischaemic heart disease, thromboembolism, stroke and the taking of antihypertensive or lipid-lowering drugs. It was confirmed for all participants the absence of any medication that might have affected the hypothalamic-pituitary-gonadal axis during the previous semester. Approval by the ethics committee of the University Hospital Dr. Peset was obtained and the study was performed in accordance with the declaration of Helsinki. All participants provided their informed consent as required by these institutions.

### Biochemical determinations

All participants were subject to an anthropometric evaluation to measure weight (kg), height (m) and waist circumference (cm) and to calculate the body mass index (BMI = weight (kg)/height (m)^2^). The weight was determined without footwear and with light clothing using electronic scales with an approximation of 0.1 kg and a capacity of up to 200 kg. The height was measured with a stadiometer with an approximation of 0.5 cm. The BMI was calculated by dividing the weight in kilograms by the square of the height in metres. The circumference of the waist was measured at the natural indentation between the 10th rib and the iliac crest and the circumference of the hips at the height of the major trochanter, using a metric tape with approximations of 0.5 cm. All measurements were made by the same nurse-study. Blood was collected from the antecubital vein on the follicular phase of the menstrual cycle or after 3 months of amenorrhea; after 12 hours of fasting. To separate serum and plasma from blood cells, samples were immediately centrifuged at 1500 g for 10 min at 4 _C. Fresh samples were used to measure biochemical parameters and the remaining aliquots were stored at −80 °C for subsequent measurement of lipidomic parameters.

Total cholesterol and triglycerides were measured using enzymatic assays (Beckman Coulter, La Brea, CA, USA). High density lipoprotein cholesterol (HDLc) levels were obtained by a direct method using a Beckman LX-20 autoanalyser (Beckman Coulter, La Brea, CA, USA). The intraserial variation coefficient was < 3.5% for all determinations. Levels of low density lipoprotein cholesterol (LDLc) were calculated using the Friedewald formula^50^. Insulin concentration was determined by means of an enzymatic luminescence technique. Glucose levels were obtained with a Dax-72 autoanalyzer using enzymatic techniques (Bayer Diagnostic, Tarrytown, NY, USA). The homeostasis model assessment of insulin resistance (HOMA-IR) was calculated using baseline glucose and insulin levels:^51^ HOMA = (fasting insulin (μU/ml) × fasting glucose (mmol/L)/22.5. Concentration of high sensitivity C-reactive protein (hsCRP) was assessed by an immunonephelometric assay (Behring Nephelometer II, Dade Behring, Inc., Newark, DE, USA) which had an intra-assay coefficient of variation of 8.7% and a sensitivity of 0.01 mg/L. Follicle-stimulating hormone (FSH) and luteinizing hormone (LH) were measured using a 2-site monoclonal non-isotopic system (Architect, Abbott Laboratories, Abbott Park, IL). Androstenedione, testosterone and sex hormone binding globulin (SHBG) were measured in our hospital’s Clinical Analysis Service using specialized chemiluminiscence techniques.

Differences among anthropometric and metabolic parameters were analyzed by paired paired t-test for equal variances (p < 0.05 with Benjamini- Hochberg Multiple Testing Correction) and differences were considered significant when p < 0.05).

### Lipidomic analysis

#### Chemicals

Synthetic lipids were from Avanti Polar Lipids Inc. (Alabaster, AL, USA) and Sigma-Aldrich (Madrid, Spain). Fatty acid methyl ester standards were from Larodan Fine Chemicals (Mälmo, Sweden) and from Sigma-Aldrich (Madrid, Spain). Methyl tert-butyl ether (MTBE) LC-MS, acetonitrile LC-MS, isopropanol LC-MS, potassium chloride, chloroform, ammonium formate and ammonium hydroxide were purchased from Sigma-Aldrich (Madrid, Spain); methanol was from Carlo Erba (Milano, Italy); acetone was from Riedel-de-Häen (Seelze, Germany); and LC/MS-grade isopropanol and formic acid were from Baker (Phillipsburg, NJ, USA).

### Untargeted lipidomic analysis: Global lipidomic profile

#### Preparation of lipid standards

For external and internal standardization lipid standards consisting of labeled lipids (see Supplementary Table 1) were used. Stock solutions were prepared by dissolving lipid standards in MTBE at a concentration of 1 mg/mL and working solutions were diluted to 2.5 μg/ mL in MTBE.

#### Lipid extraction

A lipidomic analysis was performed to the plasma sample based on a previously validated method^52^. Briefly, to precipitate the protein fraction, 5 μl of miliQ water and 20 μl of methanol were added to 10 μl of plasma sample. After the addition, samples were shaken for 2 min. Then, 250 μl of MTBE plus internal standards were added and samples were ultra-sounded in a water bath (ATU Ultrasonidos, Valencia, Spain) with a frequency and power of 40 kHz and 100 W, respectively, at 10 °C for 30 min. Then, 75 μL of miliQ water were added to the mixture and organic phase was separated by centrifugation (1,400 g) at 10 °C for 10 min. The upper phase, containing all the extracted lipid species, was collected and subjected to mass-spectrometry. A pool of all lipid extracts was prepared and used as quality controls (QC) as previously described^53^.

#### LC-MS/MS method

Lipid extracts were subjected to liquid chromatography coupled to mass-spectrometry (LC-MS) using an Agilent UPLC 1290 coupled to the Q-TOF MS/MS 6520 (Agilent Technologies, Barcelona, Spain) basing on previously published method^54^. Sample compartment was refrigerated at 4 °C and, for each sample, 10 μl of lipid extract was applied onto 1.8 μm particle 100 × 2.1 mm id Waters Acquity HSS T3 column (Waters, Mildord, MA, USA) heated at 55 °C. The flow rate was 400 μl/min with solvent A composed of 10 mM ammonium acetate in acetonitrile-water (40:60, v/v) and solvent B composed of 10 mM ammonium acetate in acetonitrile-isopropanol (10:90, v/v). The gradient started at 40% B and reached 100% B in 10 min and held for 2 min. Finally, the system was switched back to 60% B and equilibrated for 3 min. Duplicate runs of the samples were performed to collect positive and negative electrospray ionized lipid species in a TOF mode, operated in full-scan mode at 100 to 3000 m/z in an extended dynamic range (2 GHz), using N_2_ as nebulizer gas (5 L/min, 350 °C). The capillary voltage was set 3500 V with a scan rate of 1 scan/s. Continuous infusion using a double spray with masses 121.050873, 922.009798 (positive ion mode) and 119.036320, 966.000725 (negative ion mode) was used for in-run calibration of the mass spectrometer. For MS/MS analyses, we applied a previously described method^55^.

#### Data analyses

The MassHunter Data Analysis Software (Agilent Technologies, Barcelona, Spain) was used to collect the results and the MassHunter Qualitative Analysis Software (Agilent Technologies, Barcelona, Spain) to obtain the molecular features of the samples, representing different, co-migrating ionic species of a given molecular entity (i.e. ion adducts) using the Molecular Feature Extractor algorithm (Agilent Technologies, Barcelona, Spain)^31^. We selected samples with a minimum absolute abundance of 5000 counts and with a minimum of 2 ions. Compounds from different samples were aligned using a RT window of 0.1% ± 0.15 min and a mass window of 10.0 ppm ± 2.0 mDa. Only common features (found in at least 50% of the samples of the same condition) were analyzed, correcting for individual bias and excluding possible contaminants and artefacts. Finally, MassHunter Mass Profiler Professional Software (Agilent Technologies, Barcelona, Spain) was used to perform a non-targeted lipidomic analysis over the extracted features. Only common features (found in at least 50% of the samples of the same condition) were taken into account to correct for individual bias. Multivariate statistics (Hierarchical Clustering, PCA and Random Forest analyses) and biomarker analysis were done using both MassHunter Mass Profiler Professional and Metaboanalyst softwares^56,57^. The masses representing significant differences by the paired t-test for equal variances (p < 0.05 with Benjamini- Hochberg Multiple Testing Correction) were searched against the LIPID MAPS database (exact mass ppm < 20) and identified with the R-based tool LipidMatch^58^. Finally, the MS/MS spectra were checked using the LipidBlast software^59^.

### Targeted Lipidomic analysis: plasma fatty acids composition

#### Fatty acid preparation

After lipid extraction, fatty acyl groups were analyzed as methyl esters derivatives by gas chromatography (GC)^55^. Briefly, fatty acids were transesterified by incubation in 2 ml of 5% methanolic HCl at 75 °C for 90 min. The resulting fatty acid methyl esters (FAMEs) were extracted by adding 2 ml of n-pentane and 1 ml of saturated NaCl solution. The n-pentane phase was separated, evaporated under N2 gas, re-dissolved in 80 μl of carbon disulfide and 2 μl were used for GC analysis.

#### GC method

The analysis was performed on a GC System 7890A with a Series Injector 7683B and a flame ionization detector (FID) (Agilent Technologies Inc., Barcelona, Spain) equipped with a DBWAX capillary column (length 30 m × inner diameter 0.25 mm × film thickness 0.20 μm; Agilent Technologies Inc., Barcelona, Spain). The injections were performed in the splitless mode. The temperature of the injector was 220 °C. The flow rate of helium (99.99%) carrier gas was maintained at a constant rate of 1.8 ml/min. The column temperature was held at 145 °C for 5 min; subsequently, the column temperature was increased by 2 °C/min to 245 °C for 50 min, and held at 245 °C for 10 min, and with a post-run of 250 °C for 10 min.

#### Data analysis

Identification of the twenty-four FAMEs was made by comparison with authentic standards. Results were expressed as mol%. The fatty acid profile detected, identified and quantified represents more than 95% of the total chromatogram. The following fatty acid indexes were calculated: saturated fatty acids (SFA); unsaturated fatty acids (UFA); monounsaturated fatty acids (MUFA); polyunsaturated fatty acids (PUFA) from n-3 and n-6 series (PUFAn-3 and PUFAn-6); average chain length (ACL) = [(Σ%Total14 × 14) + (Σ%Total16 × 16) + (Σ%Total18 × 18) + (Σ%Total20 × 20) + (Σ%Total22 × 22) + (Σ%Total24 × 24)]/100]; double bond index (DBI) = [(1 × Σmol% monoenoic) + (2 × Σmol% dienoic) + (3 × Σmol% trienoic) + (4 × Σmol% tetraenoic) + (5 × Σmol% pentaenoic) + (6 × Σmol% hexaenoic)]; peroxidizability index (PI) = [(0.025 × Σmol% monoenoic) + (1 × Σmol% dienoic) + (2 × Σmol% trienoic) + (4 × Σmol% tetraenoic) + (6 × Σmol% pentaenoic) + (8 × Σmol% hexaenoic)]; and anti-inflammatory index (AI): [[(20:3n-6) + (20:5n-3) + (22:6n-3)]/ (20:4n-6)] * 100. Comparisons between groups were analyzed with a paired t-test for equal variances. The level of statistical significance was set at p < 0.05 in all the analyses.

## Supplementary information

Supplementary information

## Data Availability

The datasets generated during and/or analysed during the current study are available from the corresponding author on reasonable request.

## References

[1] El Hayek S, Bitar L, Hamdar LH, Mirza FG, Daoud G (2016). Poly Cystic Ovarian Syndrome: An Updated Overview. Front. Physiol..

[2] Diamanti-Kandarakis E, Dunaif A (2012). Insulin Resistance and the Polycystic Ovary Syndrome Revisited: An Update on Mechanisms and Implications. Endocr. Rev..

[3] Diamanti-Kandarakis E, Papavassiliou AG (2006). Molecular mechanisms of insulin resistance in polycystic ovary syndrome. Trends Mol. Med..

[4] Yildiz BO, Knochenhauer ES, Azziz R (2008). Impact of Obesity on the Risk for Polycystic Ovary Syndrome. J. Clin. Endocrinol. Metab..

[5] Randeva HS (2012). Cardiometabolic Aspects of the Polycystic Ovary Syndrome. Endocr. Rev..

[6] McGarry JD (1992). What if Minkowski had been ageusic? An alternative angle on diabetes. Science.

[7] Petersen KF, Shulman GI (2006). Etiology of insulin resistance. Am. J. Med..

[8] Apridonidze T, Essah PA, Iuorno MJ, Nestler JE (2005). Prevalence and Characteristics of the Metabolic Syndrome in Women with Polycystic Ovary Syndrome. J. Clin. Endocrinol. Metab..

[9] Legro RS, Kunselman AR, Dodson WC, Dunaif A (1999). Prevalence and predictors of risk for type 2 diabetes mellitus and impaired glucose tolerance in polycystic ovary syndrome: a prospective, controlled study in 254 affected women. J Clin Endocrinol Metab.

[10] Bickerton AST (2005). Cardiovascular risk in women with polycystic ovarian syndrome (PCOS). J Clin Pathol.

[11] Conway GS, Agrawal R, Betteridge DJ, Jacobs HS (1992). Risk factors for coronary artery disease in lean and obese women with the polycystic ovary syndrome. Clin. Endocrinol. (Oxf)..

[12] Ehrmann DA (2006). Prevalence and Predictors of the Metabolic Syndrome in Women with Polycystic Ovary Syndrome. J. Clin. Endocrinol. Metab..

[13] Yang C, Geng Y, Li Y, Chen C, Gao Y (2015). Impact of ovarian endometrioma on ovarian responsiveness and IVF: a systematic review and meta-analysis. Reprod. Biomed. Online.

[14] Gregor MF, Hotamisligil GS (2007). *Thematic review series: Adipocyte Biology*. Adipocyte stress: the endoplasmic reticulum and metabolic disease. J. Lipid Res..

[15] Hotamisligil GS (2010). Endoplasmic Reticulum Stress and the Inflammatory Basis of Metabolic Disease. Cell.

[16] Duleba AJ, Dokras A (2012). Is PCOS an inflammatory process?. Fertil. Steril..

[17] González F, Rote NS, Minium J, Kirwan JP (2006). Increased Activation of Nuclear Factor κB Triggers Inflammation and Insulin Resistance in Polycystic Ovary Syndrome. J. Clin. Endocrinol. Metab..

[18] Victor VM (2015). Effects of metformin on mitochondrial function of leukocytes from polycystic ovary syndrome patients with insulin resistance. Eur. J. Endocrinol..

[19] Victor VM (2009). Mitochondrial Complex I Impairment in Leukocytes from Polycystic Ovary Syndrome Patients with Insulin Resistance. J. Clin. Endocrinol. Metab..

[20] Lizneva D (2016). Criteria, prevalence, and phenotypes of polycystic ovary syndrome. Fertil. Steril..

[21] Jové M (2018). Lipidomics reveals altered biosynthetic pathways of glycerophospholipids and cell signaling as biomarkers of the polycystic ovary syndrome. Oncotarget.

[22] Wild RA, Rizzo M, Clifton S, Carmina E (2011). Lipid levels in polycystic ovary syndrome: systematic review and meta-analysis. Fertil. Steril..

[23] Mudali S (2005). Endogenous Postmenopausal Hormones and Serum Lipids: The Atherosclerosis Risk in Communities Study. J. Clin. Endocrinol. Metab..

[24] Moran LJ, Mundra PA, Teede HJ, Meikle PJ (2017). The association of the lipidomic profile with features of polycystic ovary syndrome. J. Mol. Endocrinol..

[25] Pernicova I, Korbonits M (2014). Metformin—mode of action and clinical implications for diabetes and cancer. Nat. Rev. Endocrinol..

[26] Collier CA, Bruce CR, Smith AC, Lopaschuk G, Dyck DJ (2006). Metformin counters the insulin-induced suppression of fatty acid oxidation and stimulation of triacylglycerol storage in rodent skeletal muscle. Am. J. Physiol. Metab..

[27] Sivalingam VN, Myers J, Nicholas S, Balen AH, Crosbie EJ (2014). Metformin in reproductive health, pregnancy and gynaecological cancer: established and emerging indications. Hum. Reprod. Update.

[28] Diamanti-Kandarakis E, Christakou CD, Kandaraki E, Economou FN (2010). Metformin: an old medication of new fashion: evolving new molecular mechanisms and clinical implications in polycystic ovary syndrome. Eur. J. Endocrinol..

[29] Palomba S, Falbo A, Zullo F, Orio F (2009). Evidence-Based and Potential Benefits of Metformin in the Polycystic Ovary Syndrome: A Comprehensive Review. Endocr. Rev..

[30] Wetmore JB (2014). Polycystic Kidney Disease and Cancer after Renal Transplantation. J. Am. Soc. Nephrol..

[31] Sana TR, Roark JC, Li X, Waddell K, Fischer SM (2008). Molecular formula and METLIN Personal Metabolite Database matching applied to the identification of compounds generated by LC/TOF-MS. J. Biomol. Tech..

[32] Escobar-Morreale HF (2018). Polycystic ovary syndrome: definition, aetiology, diagnosis and treatment. Nat. Rev. Endocrinol..

[33] Radosh L (2009). Drug treatments for polycystic ovary syndrome. Am. Fam. Physician.

[34] Naderpoor N (2015). Metformin and lifestyle modification in polycystic ovary syndrome: systematic review and meta-analysis. Hum. Reprod. Update.

[35] Zabielski P (2018). The effect of high fat diet and metformin treatment on liver lipids accumulation and their impact on insulin action. Sci. Rep..

[36] Holland WL, Summers SA (2008). Sphingolipids, Insulin Resistance, and Metabolic Disease: New Insights from *in Vivo* Manipulation of Sphingolipid Metabolism. Endocr. Rev..

[37] Karakas SE (2017). New biomarkers for diagnosis and management of polycystic ovary syndrome. Clin. Chim. Acta.

[38] Lucki NC, Sewer MB (2010). The interplay between bioactive sphingolipids and steroid hormones. Steroids.

[39] Santana, P. *et al*. Interleukin-IP Stimulates Sphingomyelin Hydrolysis in Cultured Granulosa Cells: Evidence for a Regulatory Role of Ceramide on Progesterone and Prostaglandin Biosynthesis*. *Endocrinology* **137** (1996). 10.1210/endo.137.6.8641202 8641202

[40] Chaurasia B, Summers SA (2015). Ceramides – Lipotoxic Inducers of Metabolic Disorders. Trends Endocrinol. Metab..

[41] Gancheva S, Jelenik T, Álvarez-Hernández E, Roden M (2018). Interorgan Metabolic Crosstalk in Human Insulin Resistance. Physiol. Rev..

[42] Aburasayn H, Al Batran R, Ussher JR, Batran AR (2016). Targeting ceramide metabolism in obesity. Am J Physiol Endocrinol Metab.

[43] Naudí A (2013). Membrane lipid unsaturation as physiological adaptation to animal longevity. Front. Physiol..

[44] Poisson J-PG, Cunnane SC (1991). Long-chain fatty acid metabolism in fasting and diabetes: relation between altered desaturase activity and fatty acid composition. J. Nutr. Biochem..

[45] Wang Y (2006). Regulation of hepatic fatty acid elongase and desaturase expression in diabetes and obesity. J. Lipid Res..

[46] Tripathy S, Torres-Gonzalez M, Jump DB (2010). Elevated hepatic fatty acid elongase-5 activity corrects dietary fat-induced hyperglycemia in obese BL/6J mice. J. Lipid Res..

[47] Yashiro H (2016). A Novel Selective Inhibitor of Delta-5 Desaturase Lowers Insulin Resistance and Reduces Body Weight in Diet-Induced Obese C57BL/6J Mice. PLoS One.

[48] Rotterdam ESHRE/ASRM-Sponsored PCOS consensus workshop group. Revised 2003 consensus on diagnostic criteria and long-term health risks related to polycystic ovary syndrome (PCOS). *Hum. Reprod*. **19**, 41–7 (2004). 10.1093/humrep/deh098 14688154

[49] Azziz R (2005). Diagnostic criteria for polycystic ovary syndrome: a reappraisal. Fertil. Steril..

[50] Friedewald, W. T., Levy, R. I. & Fredrickson, D. S. Estimation of the Concentration of Low-Density Lipoprotein Cholesterol in Plasma, Without Use of the Preparative Ultracentrifuge. *Clin. Chem*. **18** (1972). 4337382

[51] Matthews DR (1985). Homeostasis model assessment: insulin resistance and beta-cell function from fasting plasma glucose and insulin concentrations in man. Diabetologia.

[52] Pizarro C, Arezana-Rámila I, Pérez-del-Notario N, Pérez-Matute P, González-Sáiz JM (2013). Plasma Lipidomic Pro fi ling Method Based on Ultrasound Extraction and Liquid Chromatography Mass Spectrometry. Anal. Chem..

[53] Want EJ (2012). Global metabolic profiling of animal and human tissues via UPLC-MS. Nat. Protoc..

[54] Castro-Perez JM (2010). Comprehensive LC−MS E Lipidomic Analysis using a Shotgun Approach and Its Application to Biomarker Detection and Identification in Osteoarthritis Patients. J. Proteome Res..

[55] Jové M (2014). Plasma lipidomics discloses metabolic syndrome with a specific HDL phenotype. FASEB J..

[56] Xia, J. & Wishart, D. S. Using MetaboAnalyst 3.0 for Comprehensive Metabolomics Data Analysis. In *Current Protocols in Bioinformatics* 14.10.1–14.10.91 (John Wiley & Sons, Inc., 10.1002/cpbi.11 2016). 10.1002/cpbi.11 27603023

[57] Chong J (2018). MetaboAnalyst 4.0: towards more transparent and integrative metabolomics analysis. Nucleic Acids Res..

[58] Koelmel JP (2017). LipidMatch: an automated workflow for rule-based lipid identification using untargeted high-resolution tandem mass spectrometry data. BMC Bioinformatics.

[59] Kind T (2013). LipidBlast in silico tandem mass spectrometry database for lipid identification. Nat. Methods.

