# Supplementary material for: Metformin induces lipid changes on sphingolipid species and oxidized lipids in polycystic ovary syndrome women
Source: Sci Rep. 2019 Nov 5;9:16033. doi: 10.1038/s41598-019-52263-w (PMC6831788; doi:10.1038/s41598-019-52263-w)
Supplement: Supplementary file 1 — Supplementary information [file 41598_2019_52263_MOESM1_ESM.docx]

**Supplementary Table 1**. Class representative and extraction internal standards added to the plasma samples analyzed.

| **Compound** | **Reference** |
| --- | --- |
| 1,3(d5)-dihexadecanoyl-glycerol | 110537, Avanti Polar Lipids |
| 1,3(d5)-dihexadecanoyl-2-octadecanoyl-glycerol | 110543, Avanti Polar Lipids |
| 1-hexadecanoyl(d31)-2-(9Z-octadecenoyl)-sn-glycero-3-phosphate | 110920, Avanti Polar Lipids |
| 1-hexadecanoyl(d31)-2-(9Z-octadecenoyl)-sn-glycero-3-phosphocholine | 110918, Avanti Polar Lipids |
| 1-hexadecanoyl(d31)-2-(9Z-octadecenoyl)-sn-glycero-3-phosphoethanolamine | 110921, Avanti Polar Lipids |
| 1-hexadecanoyl-2-(9Z-octadecenoyl)-sn-glycero-3-phospho-(1'-rac-glycerol-1',1',2',3',3'-d5) | 110899, Avanti Polar Lipids |
| 1-hexadecanoyl(d31)-2-(9Z-octadecenoyl)-sn-glycero-3-phospho-myo-inositol | 110923, Avanti Polar Lipids |
| 1-hexadecanoyl(d31)-2-(9Z-octadecenoyl)-sn-glycero-3-[phospho-L-serine] | 110922, Avanti Polar Lipids |
| 26:0-d4 Lyso PC | 860389, Avanti Polar Lipids |
| 18:1 Chol (D7) ester | 111015, Avanti Polar Lipids |
| cholest-5-en-3ß-ol(d7) | LM-4100, Avanti Polar Lipids |
| D-erythro-sphingosine-d7 | 860657, Avanti Polar Lipids |
| D-erythro-sphingosine-d7-1-phosphate | 860659, Avanti Polar Lipids |
| N-palmitoyl-d31-D-erythro-sphingosine | 868516, Avanti Polar Lipids |
| N-palmitoyl-d31-D-erythro-sphingosylphosphorylcholine | 868584, Avanti Polar Lipids |
| Octadecanoic acid-2,2-d2 | 19905-58-9, Sigma Aldrich |
